# Money lies and extramarital ties: Predicting separate and joint occurrences of financial deception and extramarital infidelity

**DOI:** 10.3389/fpsyg.2022.1038169

**Published:** 2022-11-22

**Authors:** Jeffrey P. Dew, Matthew T. Saxey, Alison Mettmann

**Affiliations:** ^1^School of Family Life, Brigham Young University, Provo, UT, United States; ^2^Department of Mathematics Education, Brigham Young University, Provo, UT, United States

**Keywords:** commitment, extramarital infidelity, marital financial deception, relationship satisfaction, affair

## Abstract

**Introduction:**

Although spouses frequently financially deceive each other (MFD; i.e., marital financial deception), few studies have examined this relationship behavior. The purpose of our study is to examine predictors of separate and joint occurrences of MFD and extramarital affairs (EMI). We chose the predictors we tested using social exchange theory (SET).

**Methods:**

We used a national sample of married individuals and multinomial logistic regression analyses to examine how different predictors were associated with membership in three different groups (MFD with no EMI, EMI with no MFD, and both MFD and EMI) relative to the group of participants who reported neither behaviors.

**Results:**

Relationship satisfaction was associated with a lower likelihood of being in the MFD-only group, moral commitment was negatively associated with membership in both EMI groups, and personal dedication commitment was negatively associated with membership in both MFD groups. Flirting with someone other than one’s spouse was positively associated with being in all three groups relative to the reference group. The personal importance of religion was not associated with group membership.

**Discussion:**

Moral commitment, personal dedication commitment, and flirting with someone other than one’s spouse predicted these two types of marital deception. It is likely that other issues that affect marital outcomes, comparisons, and monitoring alternatives to the relationship may predict MFD and/or EMI.

## Introduction

Although spouses frequently financially deceive each other (MFD; i.e., marital financial deception), few studies have examined this relationship behavior. Estimates of those who engage in MFD range from 40 to 60% in national samples (National Endowment for Financial Education, 2018; Trujillo et al., 2019); by way of contrast, very few peer-reviewed studies of MFD have been published (Junare and Patel, 2012; Jeanfreau et al., 2018, 2020; Dew et al., 2022). Thus, a common marital behavior that likely has negative relationship consequences nevertheless remains relatively unexamined by scholars.

Extramarital infidelity (EMI; i.e., having sexual intercourse with someone who is not one’s spouse and without the knowledge and/or consent of one’s spouse) is another maritally destructive behavior that involves deception. Unlike MFD, however, scholars have studied EMI in great depth. Researchers have found that predictors of EMI can be found within individuals, within marital relationships, and outside marital relationships (see Atkins et al., 2001; Allen et al., 2005; Whisman et al., 2007; Fincham and May, 2017 for reviews). For example, having been sexually unfaithful in the past, being dissatisfied with the relationship, and having lower commitment to the marriage are all associated with greater likelihoods of infidelity (Fincham and May, 2017). Contrastingly, many aspects of religiosity, such as prayer and religious worship service attendance are associated with lower probabilities of sexual infidelity (Fincham and May, 2017).

Little is known, however, about the association between EMI and MFD. For example, deceiving one’s spouse financially may prime one to betray them sexually. Alternatively, individuals who are already cheating on their spouse may use MFD to facilitate or maintain the affair (e.g., by buying gifts for a paramour). Furthermore, while many married individuals report engaging in some form of MFD (National Endowment for Financial Education, 2018), only 15–17% of ever married individuals report having engaged in EMI (Wilcox et al., 2019). Consequently, MFD does not necessarily lead to or follow from EMI. Rather, the relationship between these two forms of marital betrayal seems more complex. In this study, we used a national sample of married individuals to identify predictors of participants reporting MFD behaviors alone, EMI behaviors alone, or reporting both types of marital deception relative to those who reported engaging in neither type of deception.

This study expands the body of literature on marriage and on relationship deception in multiple ways. To our knowledge, this is the first study that tests possible predictors of the joint occurrence of MFD and EMI behaviors. This contributes to the growing body of literature that is examining the simultaneous sexual and the financial aspects of relationships. Additionally, we answer questions about marital deception that occurs across domains instead of simply examining multiple forms of the same deception (e.g., sexual infidelity). A final addition to the literature is that we examine these predictors using national data. Only one of the four MFD studies we mentioned use national data, so this study may generalize better than the previous work.

Understanding predictors of these maritally deceptive behaviors is important for multiple reasons. First, MFD and EMI can be relationally destructive—EMI especially so (Previti and Amato, 2004; Trujillo et al., 2019). Further, by studying MFD and EMI together, we can better understand how these two processes function jointly and separately. Finally, given that MFD and EMI might come up as issues in therapeutic settings, understanding them more may help practitioners as they work with married couples.

## Marital financial deception and extramarital infidelity in social exchange theory

### Social exchange theory

Social exchange theory (SET) originated in social psychology’s interpersonal relationship area. It is an economic, exchange-based theory. SET suggests that relationship rewards, costs, expectations, and alternatives interact to entice individuals to remain in, modify, or leave their relationships (Thibault and Kelley, 1959).

Nye (1979) formalized the SET process that married individuals go through as they evaluate their relationships. First, each spouse evaluates the actual costs and benefits they receive from their marriage. The costs and benefits that each spouse experiences is termed “outcomes” in SET. Next, each spouse compares their actual marital outcomes to those they expect to receive. Appropriately, these expected marital outcomes are termed “the comparison level” or “CL” in SET.

The comparison of actual outcomes to the CL determines whether each individual spouse is satisfied or dissatisfied with the marriage (Thibault and Kelley, 1959; Nye, 1979). If the marital outcomes a spouse receives equal or exceed the CL, then that spouse will be satisfied with the relationship. In this situation, the marital relationship is giving the spouse at least what they expect from it. SET suggests that satisfied spouses will not move on in the process. Rather, they will remain satisfied until their outcomes and/or their expectations change.

However, if a spouse’s outcomes fall beneath their CL, they will become dissatisfied with the relationship and will continue on in the process. That is, SET suggests that receiving less than what one desires from a relationship will lead to dissatisfaction (Thibault and Kelley, 1959; Nye, 1979). Once an individual spouse becomes dissatisfied, they are faced with many potential courses of action. These options include attempting to change the relationship so they can realize the outcomes they desire, seeking desired outcomes through means outside of the relationship, living in the relationship with dissatisfaction, lowering the comparison level so that they become satisfied, and leaving the relationship.

The next step in the SET process is that relationally dissatisfied individuals will compare their current outcomes with outcomes they feel they would receive in relationship alternatives. The subjective outcomes they believe they can realize in other relationship situations are called the comparison level of the alterative (CL_alt,_ Nye, 1979). If their present outcomes exceed the CL_alt_ then they will remain in the relationship and try to realize better outcomes. If, however, the CL_alt_ (i.e., the outcomes they expect to gain outside their marriage) exceeds their present outcomes, they will leave the relationship.

### Marital betrayal in social exchange theory

Based on this SET process, we assert that spouses engage in MFD and EMI to obtain something they are not getting in their present relationship, to modify relationship dynamics, and/or to end their relationship. For example, some research suggests that individuals may pursue infidelity for reasons of a lack of love in their primary relationship or to find greater variety (Selterman et al., 2021). Furthermore, one spouse who feels that the other spouse is too “miserly” might engage in MFD to obtain desired goods and services without their spouse’s knowledge and/or to avoid marital conflict (Jeanfreau et al., 2020). Consequently, we expect that predictors of MFD and EMI will be factors that influence spouses’ outcomes, expectations, the attractiveness of alternatives, or a combination of these factors.

### Moral commitment as expressing an expectation

Moral commitment to one’s marriage (i.e., the sense of a moral obligation to continue in a marriage, uphold marital norms/vows, etc.; Johnson et al., 1999) might also negatively predict EMI and, possibly, MFD. Most Americans report that EMI is “always wrong” (Wilcox et al., 2019, p. 4). Consequently, it seems that most Americans *expect* marital fidelity not only from spouses but from themselves. They want to behave in a way that upholds marital norms and/or their wedding vows to their spouse. These attitudes might then guide their behavior.

Specifically, research has demonstrated an association between moral commitment and EMI. One study showed that those with a less strict definition of what constituted “cheating” in their marriage were over twice as likely to report engaging in EMI than those who held strict definitions of marital cheating (Dew, 2020). Likewise, morally valuing marriage as an exclusive relationship likely acts as a barrier to EMI (Jeanfreau and Mong, 2019). Despite this evidence, scholars have rarely examined moral commitment as a predictor of EMI, so this study stands to contribute toward understanding moral commitment’s efficacy as a predictor of EMI.

Researchers have also not yet examined the association between moral commitment and MFD. However, we feel it reasonable to assume that a negative association may exist. For example, it stands to reason that those with stricter definitions of marital fidelity might also avoid MFD because they could view this behavior as “unfaithful” or “deceitful” (Jeanfreau et al., 2020). Personally held expectations of moral commitment, then, may strengthen spouses’ resolve to forego MFD.

*H1*: Moral commitment will be negatively associated with EMI and MFD.

### Flirting as evaluating or seeking alternatives to the relationship

SET asserts that if a married individual is exploring alternative relationships (e.g., joining a dating app while married, flirting with co-workers), this suggests that they are already dissatisfied in their marriage. In a United States nationally representative sample, for example, a negative correlation between engaging in online unfaithful behaviors and marital happiness arose (Wilcox et al., 2019).

Furthermore, exploring possible alternative partner options may indicate that an individual is willing to leave the marriage. In their study, Wilcox et al., (2019) found that an individuals’ perceptions of the stability of their marriage was inversely associated with engaging in online unfaithful behaviors. For example, qualitative research suggests that flirtatious behavior is one indicator if someone wants to have a marital affair (Jeanfreau and Mong, 2019). Clinicians also suggest that infidelity begins with flirting (Levine, 2005). Thus, reporting having flirted with others while one is married is likely to predict EMI because in SET, flirting is a strong indicator that one might be looking to leave their marriage; they are actively evaluating alternatives.

We are not aware of any studies that have examined the association between flirting with someone other than one’s spouse and MFD. Allen et al. (2005) suggested that marital betrayal progresses in stages with “smaller” types of betrayal occurring first. Consequently, flirting with someone other than one’s spouse, already an indicator of an unhappy marriage, might make it more likely that a spouse might engage in MFD (i.e., another “minor” instance of unfaithfulness). Therefore, flirting with someone other than one’s spouse might play a role in predicting EMI and/or MFD.

*H2*: Reports of flirting with someone other than one’s spouse will be positively associated with EMI and MFD.

### Marital satisfaction across the SET process

As noted above, SET suggests that individual spouses who receive outcomes that at least meet their expectations, are those who become maritally satisfied. In these satisfied marriages, the valence of outcomes to desires is at least even, if not positive. SET asserts that because of this, those who are satisfied in their marriage will be less likely to notice alternatives to the marriage or will devalue them if they do. Research has confirmed this theoretical assertion. Among newlywed couples, for example, marital sexual satisfaction was negatively associated with participants’ views of potential alternative partners to the relationship (Stanik and Bryant, 2011). In a classic study of the questions, researchers found a negative association between relationship satisfaction and participants’ attractiveness ratings of potential alternative partners (Johnson and Rusbult, 1989). Simply put, individuals who are satisfied in their marriage have less reason to evaluate alternatives to their relationship than do dissatisfied individuals.

Even if an opportunity arises to betray one’s spouse, a happily married individual is less likely to do so because they will risk a lot—a marriage that is giving them at least what they believe they should receive. Research has shown that marital satisfaction is negatively associated with EMI (Fincham and May, 2017). It has also shown that marital satisfaction is negatively associated with MFD (Jeanfreau et al., 2018).

If marital satisfaction is low, SET asserts that the marriage is not meeting expectations; this might promote MFD and/or EMI so that one or both spouses can be happier. As noted in the previous paragraph, marital satisfaction is indeed a barrier to both MFD and EMI. Furthermore, a study conducted on a sample of married and cohabiting couples in Brazil found that participants cited relationship unhappiness as the top reason for engaging in EMI (Scheeren et al., 2018). It is also the case that couples who do not share financial values report lower levels of marital quality in comparison to those couples who do share financial values (Baisden et al., 2018). When values are not shared, partners may be more likely to participate in financial activities the other disapproves of, whether secretly or explicitly, likely leading to increased conflict and lower relationship quality (Baisden et al., 2018). Additionally, marital satisfaction negatively predicted minor instances of infidelity (Shackelford et al., 2008; McDaniel et al., 2017), positive attitudes toward infidelity (Isma and Turnip, 2019), and EMI (Whisman et al., 2007; Shackelford et al., 2008).

*H3*: Marital satisfaction will be negatively associated with EMI and MFD.

### Personal dedication commitment across the SET process

Personal dedication to one’s marriage (i.e., the desire to invest in one’s marriage and make it work out regardless of obstacles) is a type of commitment (Stanley and Markman, 1992). Yet, personal dedication is a type of commitment separate from moral commitment. That is, personal dedication has less to do with social norms and promises, like moral commitment, and more to do with individual desires to invest in one’s marriage and/or the happiness of one’s spouse (Stanley and Markman, 1992; Johnson et al., 1999).

Personal dedication likely influences the interplay of the comparison between outcomes, the CL, and the resultant level of marital satisfaction. Deciding to invest in one’s marriage and to see it succeed despite obstacles likely entails viewing one’s marriage through a long-term lens. Personal dedication, then, may make it less likely for marital dissatisfaction to occur when outcomes fall below the CL. Thus, a spouse with high levels of personal dedication might know or hope that they will be able to overcome marital difficulties in the long run even if they are currently dissatisfied with the relationship. Personal dedication may also make it less likely that a dissatisfied spouse will evaluate the alternatives to the marriage and/or more likely to ignore alternatives even if they are aware of them.

Personal dedication is a strong predictor of various marital outcomes. For example, a *lack* of personal dedication commitment in a marriage was identified as the top reason couples ended up divorcing (Scott et al., 2013). This suggests that couples place a high value on personal dedication commitment in marriage or that a marriage is difficult to sustain once one or both spouses lose their personal dedication. Furthermore, qualitative research suggests that marital commitment, including personal dedication commitment, can be a barrier to committing infidelity (Jeanfreau and Mong, 2019). Recent quantitative research about MFD and EMI supports this qualitative finding. For MFD, personal dedication commitment—in the form of marital stability and trust of one’s partner—is negatively associated with MFD (Dew et al., 2022). Scholars have also found that personal dedication commitment predicts a lower level of sexual unfaithful behaviors (Shaw et al., 2013; Rodrigues et al., 2017).

*H4*: Personal dedication commitment will be negatively associated with EMI and MFD.

### Religiosity across the SET process

Although higher religiosity can lead to negative couple outcomes for some (Kelley et al., 2020), religiosity might contribute to better relationship outcomes as well. That is, because most religions hold marriage to be a sacred or special relationship, religious spouses may attempt to please each other (or the divine) more than married individuals who are not religious and, thus, raise the outcomes of the marriage. Religiosity positively predicts marital satisfaction (Goddard et al., 2012; Rose et al., 2021), quality (Ellison et al., 2011), and sexual satisfaction (Dew et al., 2020). A possible explanation for these findings is that religiosity is correlated with viewing a marriage as *sanctified* (i.e., having a divine character or sacred significance; Ellison et al., 2011). Because higher levels of religiosity might enhance marital satisfaction, it may also lower the likelihood of MFD and/or EMI (Jeanfreau et al., 2018; Jeanfreau and Mong, 2019).

Greater religiosity may also prevent individuals from engaging in MFD and/or EMI for two reasons. First, religious individuals likely will not want to violate something they hold to be sacred; that is, religiosity can raise the cost of marital betrayal as an alternative to the relationship. Indeed, qualitative evidence suggests that religiosity promotes marital fidelity (Dollahite and Lambert, 2007). Quantitative evidence supports the qualitative evidence—religiosity is negatively associated with EMI (Whisman et al., 2007; Whisman and Snyder, 2007; Tuttle and Davis, 2015; DeRose et al., 2021). The second reason that religiosity may reduce marital betrayal is that individuals embedded within a religious community are likely to enforce the norms of that community. Thus, individuals who regularly attend worship services may monitor others, and be monitored by others, for any religiously aberrant behavior, including marital betrayal. Thus, personal religiosity and worship service attendance might negatively predict EMI and/or potentially more minor instances of unfaithfulness like MFD.

*H5*: Personal religious importance and religious worship service attendance will be negatively associated with EMI and MFD.

## Materials and methods

### Data and sample

The data we used comes from the iFidelity data set. The iFidelity data was collected to examine contemporary attitudes and behaviors vis-à-vis fidelity in adult romantic relationships. The survey research firm YouGov collected the data in late 2019 using their national US YouGov panel of participants. Anyone on the panel who resided in the US was eligible to participate. YouGov matched the respondents to a sampling frame meant to mirror the 2016 American Community Survey (ACS). The matching resulted in a data set of 2,000 participants. YouGov then created post-stratifications weights so that when the sample was weighted, it would be nationally representative with respect to gender, age, 2016 presidential vote choice, race/ethnicity, and education.

For the purposes of our study, participants had to have reported that they were currently married. We restricted our study sample to these participants because they were the only participants in the iFidelity data who were asked if they had engaged in MFD and EMI. The requirement yielded an overall sample of 946 participants for this particular study.

### Variables

#### Dependent variable

The purpose of this study was to test variables that might predict whether participants report engaging in MFD but not EMI, EMI but not MFD, or both behaviors relative to reporting neither behaviors. Participants in the iFidelity survey self-reported their own MFD and EMI behaviors. We considered participants to have engaged in MFD behaviors if they responded affirmatively to at least one of seven questions about participating in different types of MFD (e.g., hiding a bank account/credit card/loan from their spouse; lying to one’s spouse about the cost of a purchase). We considered participants to have engaged in EMI behaviors if they affirmed that they had had anal, oral, or vaginal sex with someone other than their spouse and without their spouse’s knowledge and approval.

Some could be rightly concerned that participants might be unwilling to report on their own marital betrayals. However, 52% of participants in the iFidelity survey self-reported their own MFD behavior. Furthermore, 14.5% self-reported EMI behavior. The MFD estimate is higher than another national survey of MFD in which 40% of participants reported MFD (National Endowment for Financial Education, 2018). The estimate of EMI, though, aligns with other national data sets. For example, 15% of ever-married participants in the 2018 General Social Survey (GSS) reported EMI behavior (Wilcox et al., 2019). Thus, even if some underreporting of these behaviors occurred in the iFidelity survey, estimates are comparable to other nationally-representative surveys. Our sample is unlikely to be marked by severe underreporting of marital betrayals.

Our dependent variable was trichotomized. One value represented that the participant reported only engaging in MFD behavior (43.5% of our sample). Another value represented that the participant engaged only in EMI behavior (5.5% of the sample). The final category was engaging in both behaviors (9% of the sample). The reference, or comparison, category was the group of participants who did not report ever having engaged in MFD or EMI (42% of the sample).

#### Independent variables

##### Relationship satisfaction

The iFidelty survey measured relationship satisfaction by asking about participants’ global relationship happiness. The question specifically asked, “Taking things all together, how would you describe your current relationship.” Participants could answer 1 (*Very unhappy*) to 5 (*Very happy*).

##### Moral commitment

We operationalize moral commitment by assessing whether participants had strong personal definitions of what constituted “cheating.” We created a dichotomous variable (0 = *Average or lower definition of cheating*; 1 = *A strong definition of cheating*). The iFidelity survey asked participants whether they thought nine different behaviors constituted “cheating.” The behaviors ranged from “following an old flame online” to “having had anal, oral, or vaginal sex with someone other than one’s spouse without their spouse’s knowledge and consent.” More than half of the participants identified six of the nine behaviors as “cheating.” Three of the behaviors garnered less than 50% of agreement. Consequently, if participants labeled seven or more behaviors as “cheating” we rated them as having a strong definition of cheating.

##### Flirting

The iFidelity survey also asked participants about a range of different behaviors. It specifically asked participants whether they had flirted with someone other than their spouse. We created a dichotomous variable for the analysis (0 = *Did not report extramarital flirting*, 1 = *Reported extramarital flirting*).

##### Personal dedication commitment

We measured personal dedication commitment using three items from the commitment inventory (Stanley and Markman, 1992). The three items asked, “I want this relationship to stay strong no matter what rough times we may encounter,” “My relationship with my spouse is more important to me than almost anything else in my life,” and “I like to think of my spouse and me more in terms of ‘us’ and ‘we’ than ‘me’ and him/her.’” The response set for each of the three variables ranged from 1 (*Strongly disagree*) to 5 (*Strongly agree*). We took the mean of these items to create the personal dedication commitment variable we used in the analysis. Cronbach’s alpha for the three variables was 0.87.

Our final main independent variables were personal importance of religion and religious worship service attendance. The iFidelity survey asked participants how important religion was to them personally. Participants could respond from 1 (*Very important*) to 4 (*Not at all important*). We reverse coded the variable so that higher scores represented greater personal importance. The iFidelity survey also asked participants how frequently they attended religious worship services, on average. The response set ranged from 1 (*More than once a week*) to 6 (*Never*). We reverse coded this variable.

##### Control covariates

We also controlled for age, education, total household income, and race/ethnicity in the multinomial logistic regression. We added these variables because previous studies have shown that they are associated with EMI or because studies of their association with EMI have yielded mixed results (Fincham and May, 2017).

Participants self-reported their age in years. We used two dummy-coded variables to represent education—having a high school degree or less and having a four-year degree or more. The reference category was having some college or an associate degree. Participants reported their total household income on a scale that ranged from 1 (*Less than $10,000*) to 16 (*$500,000 or more*). Participants reported their own race and ethnicity. We used dichotomous variables to represent three race/ethnicity groups—Black non-Hispanic, Hispanic, and other race/ethnicity. The reference category is White non-Hispanic.

### Analysis

We used multinomial logistic regression to estimate our model. Multinomial logistic regression facilitates examining the association with a set of predictors and a categorical outcome variable that has more than two possible distinct responses. In this study, participants could be in one of four discreet groups that simultaneously measured participation in MFD and in EMI.

Like binary logistic regression, multinomial logistic regression compares the likelihood of being in one group relative to another based on the independent variables in the model. In the case of multinomial logistic regression, however, there are simply more comparisons because there are more possible outcome groups. These comparisons all run in the same analysis. This means that one can examine how any specific independent variable influences the odds of being in the different groups relative to the comparison group. As noted above, our reference or comparison group was those who reported neither MFD or EMI. Thus, the multivariate logistic regression estimated the probability of being in a group with MFD but with no EMI relative to being in the group who reported neither, the probability of being in a group without MFD but with EMI relative to being the group with neither, and the probability of being in a group reporting both MFD and EMI relative to being in the group reporting neither behavior.

Only three of the variables had missing responses. This included relationship satisfaction (*n* = 2 missing), personal dedication commitment (*n* = 3 missing) and total household income (*n* = 128 missing). Instead of list-wise deleting these cases, we used mean imputation to fill the missing values. Although multiple imputation and FIML are generally better ways to handle missing data than mean imputation, the first two variables had so few missing that we felt that mean-imputation was sufficient. Furthermore, because income was not statistically significant in any of the models, we concluded after the analysis that mean imputation was sufficient.

We found that personal religious importance and religious worship service were highly correlated (*r* = 0.72, *p* < 0.001). When they were both in the model, their signs were opposite with group membership relative to what they were in bivariate associations. We suspected that high levels of multicollinearity between these two variables in the multivariate analysis. Consequently, we dropped the worship service attendance variable from the model and used only personal religious importance.

## Results

### Descriptive results

The statistics describing the sample are in Table 1. Participants reported a relatively high level of relationship satisfaction in their marriages. The mean was 4.24 out of a possible 5 points. Another 49% of participants reported having a strong definition of cheating. Given that we dichotomized the variable using a mean split, this is not surprising. Participants who reported flirting with someone other than their spouse while married composed 32% of the data. Like relationship satisfaction, the mean of personal dedication commitment was relatively high (4.4 of 5). Finally, participants reported a mean score of 2.96 (out of 4) on the personal importance of religion variable. Table 1 also shows the demographic characteristics of the study’s participants.

**Table 1:** Descriptive statistics.

	Mean or %	SD	Minimum–Maximum
Relationship satisfaction	4.24	0.93	1–5
Strong definition of cheating (moral commitment)	49%		
Flirting with someone other than one’s spouse	32%		
Personal dedication commitment	4.40	0.84	1–5
Personal importance of religion	2.96	1.14	1–4
Age	52.04	15.43	18–89
High school degree or lower	37%		
Four-year college degree or higher	36%		
Total household income	6.79	3.14	1–16
Black	7.9%		
Hispanic	11.4%		
Other race/ethnicity	8.6%		

### Multivariate results

Table 2 shows the results of the multinomial logistic regression analysis. These results were obtained with only one analysis. However, we discuss the likelihood of being a member in each group separately to facilitate clarity. The order in which we discuss the multivariate results is the same as the order of Table 2, moving from left to right.

**Table 2:** Multinomial logistic regression predicting marital financial deception/extramarital infidelity group membership.

	Group 1 marital financial deception, no extramarital infidelity^a^			Group 2 no marital financial deception, extramarital infidelity^a^			Group 3 marital financial deception and extramarital infidelity^a^		
	*b*	SE_b_	Log-Odds	*b*	SE_b_	Log-Odds	*b*	SE_b_	Log-Odds
Intercept	2.99***	0.60		−1.55	1.52		2.78**	1.08	
Relationship satisfaction	−0.22*	0.10	0.80	−0.41	0.25	0.66	−0.29	0.19	0.75
Strong definition of cheating (moral commitment)^b^	−0.09	0.08	0.91	−0.71**	0.26	0.49	−0.45**	0.17	0.64
Flirting with someone other than one’s spouse^c^	0.40***	0.09	1.49	1.16***	0.23	3.19	1.73***	0.20	5.58
Personal dedication commitment	−0.33**	0.12	0.72	−0.02	0.30	0.98	−0.66**	0.21	0.52
Personal importance of religion	0.01	0.07	1.01	−0.27	0.17	0.76	0.09	0.14	1.09
Age	−0.01	0.01	0.99	0.04*	0.01	1.04	−0.01	0.01	0.99
High school degree or lower^d^	−0.01	0.10	0.99	−0.10	0.25	0.90	0.14	0.18	1.15
Four-year college degree or higher^d^	−0.22*	0.10	0.80	−0.40	0.26	0.67	−0.48*	0.20	0.62
Total household income	−0.01	0.03	0.99	−0.07	0.08	0.93	−0.02	0.06	0.98
Black^e^	−0.33*	0.14	0.72	−0.61	0.52	0.54	−0.19	0.26	0.83
Hispanic^e^	−0.22	0.12	0.80	−0.03	0.42	0.97	0.34	0.21	1.40
Other race/ethnicity^e^	0.32*	0.14	1.38	0.64*	0.32	1.90	−0.01	0.38	0.99

^a^Relative to those reporting no marital financial deception and no extramarital infidelity. ^b^Relative to having an average or below average definition of cheating. ^c^Relative to not reporting flirting with someone other than one’s spouse. ^d^Relative to having had some college or an associate’s degree. ^e^Relative to White, non-Hispanic participants. *p < 0.05, **p < 0.01, ***p < 0.001.

#### Marital financial deception without extramarital infidelity

We called the participants who reported MFD but not EMI “Group 1.” Relationship satisfaction was negatively associated with being in Group 1 (*b* = −0.22, *p* < 0.05, Log-odds = 0.80), relative to the comparison group (i.e., those who reported neither MFD nor EMI). That is, every step increase in relationship satisfaction lowered the odds of being in the MFD/no EMI group by 20%. By way of contrast, flirting with someone who is not one’s spouse was positively associated with being in Group 1 (*b =* 0.40, *p* < 0.001, Log-odds = 1.49), relative to the comparison group. Because the measure was dichotomous, reporting having flirted with someone other than one’s spouse was associated with a 49% increase in the odds of being in Group 1 compared to the reference category. Finally, both personal dedication commitment (*b* = −0.33, *p* < 0.05, Log-odds = 0.72), having a four-year college degree or higher (*b* = −0.22, *p* < 0.05, Log-odds = 0.80) and reporting Black race (*b* = −0.33, *p* < 0.05, Log-odds = 0.72) were negatively associated with Group 1 membership relative to the reference group. Reporting an “other” race ethnicity was positively associated with Group 1 membership (*b* = 0.32, *p* < 0.05, Log-odds = 1.38).

#### Extramarital infidelity without marital financial deception

We titled the participants who reported EMI but not MFD “Group 2.” The comparison group was, again, participants who reported neither MFD nor EMI. Four variables statistically predicted differences between the comparison group and Group 2. Having a strong definition of cheating (*b* = −0.71, *p* < 0.01, Log-odds = 0.49) was negatively associated with Group 2 membership. Flirting with someone other than one’s spouse (*b* = 1.16, *p* < 0.001, Log-odds = 3.19), age (*b* = 0.04, *p* < 0.05, Log-odds = 1.04) and reporting “other” race/ethnicity (*b* = 0.64, *p* < 0.05, Log-odds = 1.90), were positively associated with being in Group 2 compared to the reference group. Having a strong definition of what constituted cheating lowered odds by 51%. Flirting with someone other than one’s spouse raised the odds of being in Group 2 by 219% relative to being in the comparison group. For age and “other” race ethnicity the increases in log odds were 4% and 90$, respectively.

#### Marital financial deception with extramarital infidelity

The final group of participants, those who reported both MFD and EMI, we called “Group 3.” As with the other two analyses, the comparison group was the group of participants who reported neither MFD nor EMI. Having a strong definition of cheating (*b* = −0.45, *p* < 0.01, Log-odds = 0.64) and personal dedication commitment (*b* = −0.66, *p* < 0.01, Log-odds = 0.52) were negatively associated with being in Group 3 relative to the comparison group. Having a strong definition of what constituted cheating lowered the odds of being in Group 3 by 36%, and each step increase in personal dedication commitment was associated with 48% lower odds of being in Group 3. Finally, flirting with someone other than one’s spouse was positively associated with being in Group 3 (*b* = 1.73, *p <* 0.001, Log odds = 5.64) relative to being in the comparison group. Those who reported flirting with someone other than their own spouse had odds that were 464% higher of being in Group 3 than those who did not report flirting with someone other than one’s spouse.

We created Table 3 to summarize the findings and makes the patterns across groups and variables easier to understand.

**Table 3:** Summary of findings.

	Group 1 marital financial deception, no extramarital infidelity^a^	Group 2 no marital financial deception, extramarital infidelity^a^	Group 3 marital financial deception and extramarital infidelity^a^
Hypothesis 1: Relationship satisfaction	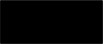		
Hypothesis 2: Strong definition of cheating (moral commitment)^b^		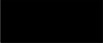	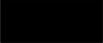
Hypothesis 3: Flirting with someone other than one’s spouse^c^	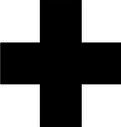	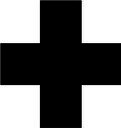	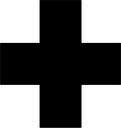
Hypothesis 4: Personal dedication commitment	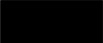		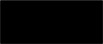
Hypothesis 5: Personal religiosity			

^a^Relative to those reporting no marital financial deception and no extramarital infidelity. ^b^Relative to having an average or below average definition of cheating. ^c^Relative to not reporting flirting with someone other than one’s spouse.

## Discussion

Theoretically informed by SET (Thibault and Kelley, 1959; Nye, 1979), we examined five predictors (i.e., relationship satisfaction, personal dedication commitment, moral commitment, flirting with someone other than one’s spouse, and personal religious importance) of participants reporting MFD behaviors alone, EMI behaviors alone, or reporting both types of marital deception relative to those who reported engaging in neither type of deception. We found support for four of our five hypotheses.

Relationship satisfaction was associated with group membership. Specifically, a one unit increase in relationship satisfaction predicted odds that were 20% lower of engaging in MFD without EMI relative to those who reported engaging in neither MFD nor EMI. Relationship satisfaction’s negative association with MFD aligned with previous findings (Jeanfreau et al., 2018; Trujillo et al., 2019; Saxey et al., 2022). However, the fact that relationship satisfaction did not predict EMI when the other variables were in the model goes contrary to other studies. (Whisman et al., 2007; Shackelford et al., 2008).

Although we cannot test the mechanism behind this association using the iFidelity data, SET provides some potential reasons for it. According to SET, individuals who are satisfied in their marriages have no reason to engage in MFD because they are already receiving what they desire from the relationship. This raises the value of their marriage, as well as the cost of engaging in behaviors that might threaten it such as MFD. The fact that relationship satisfaction failed to predict group membership in any of the EMI groups might suggest that some of the other variables in the model account for the association that other studies have found. Future research is needed to examine relationship satisfaction and the joint occurrence of MFD and EMI more closely.

Our hypothesis regarding flirting with someone other than one’s spouse was also supported. Having flirted with someone other than one’s spouse predicted a 49% higher likelihood of having engaged in MFD without EMI, a 219% higher likelihood of having engaged in EMI without MFD, and a 458% higher likelihood of having engaged in MFD and EMI relative to being in the no MFD/EMI group and relative to those who did not report extramarital flirting.

SET would assert that these extraordinarily potent effect sizes emerged because individuals who are flirting with others outside their marriage are not particularly keen on their marriage. They are already maritally dissatisfied (i.e., their outcomes are below the CL) and are at least evaluating alternative relationship options (i.e., they are comparing their current outcomes to the CLalt) if not actively seeking to leave the relationship. If a spouse flirted with alternative partners to gain something they are not getting in their marriage, it may have engaged in EMI with the person with whom they flirted (Levine, 2005; Jeanfreau and Mong, 2019). Additionally, having engaged in more “minor” instances of infidelity like extramarital flirting make it more likely to have engaged in other “minor” instances of deception like MFD (Allen et al., 2005). Consequently, SET would suggest that it should not at all be surprising that individuals that have flirted with someone other than the past are at high risk for engaging in marital betrayal. Indeed, our analysis of extramarital flirting’s association of MFD and EMI provided a strong test of SET.

This finding is notable, in part, because scholars have not quantitively examined the association between flirting with alternative partners and the likelihood of engaging in MFD and EMI. Although qualitative evidence suggests EMI begins with flirting (Jeanfreau and Mong, 2019), and clinicians suggest the same (Levine, 2005), scholars have rarely, if ever, quantitaively examined whether and how flirting with an alternative partner predicts EMI (e.g., Dew et al., 2021) and MFD. In this way, this study makes a novel contribution to the literature. Future research might profitably continue to explore the role flirting—perhaps including online flirting—might play in predicting MFD and/or EMI.

Although not as strong as our findings with flirting with someone other than one’s spouse, commitment predicted joint and separate occurrences of MFD and EMI. Although our hypotheses regarding both moral commitment and personal dedication commitment were supported, the patterns that emerged differed. Moral commitment seemed predictive of EMI. That is, moral commitment negatively predicted members in the two EMI groups (Groups 2 and 3) relative to the comparison group. This coincides with previous literature (Jeanfreau and Mong, 2019; Dew, 2020). Personal dedication commitment, by way of contrast, was negatively associated with the groups involving MFD (Groups 1 and 3).

SET did not lead us to expect these somewhat contrasting commitment findings so we can only speculate on why they occurred. Part of the differences may stem simply from measurement issues. We operationalized moral commitment as whether participants had strong definitions of what constituted “cheating.” All the measures on which this was based were measures of emotional and sexual infidelity. Thus, it should not be surprising that our measure of moral commitment was strongly associated with group membership in the EMI groups. We may have had different findings if the iFidelity survey had items involving moral commitment that were broader than attitudes toward emotional and sexual infidelity. This measurement issue is the simplest explanation for finding differences between moral and personal dedication commitment.

But the findings may have also occurred because of the differences in the two types of commitment. Personal dedication commitment indicates the extent to which an individual wants to invest in their marriage. Moral commitment measures how moral obligated an individual feels to their marriage. Interestingly, financial deception in romantic relationships has been negatively linked to not just relationship *satisfaction* (Jeanfreau et al., 2018) but also relationship *flourishing* (Saxey et al., 2022). That is, individuals in marriages that are growing in positive ways are less likely to engage in MFD. The Saxey et al. (2022) finding is, thus, similar to the finding in our study. If MFD does not register as some sort of betrayal of a spouse in the minds of married individuals, however, than moral commitment may not prevent it. Future research is necessary to resolve these questions.

We were surprised by finding no support for the association between personal religious importance and group membership. Despite evidence suggesting an association between religiosity and EMI (Dollahite and Lambert, 2007; Whisman et al., 2007; Whisman and Snyder, 2007; Tuttle and Davis, 2015; DeRose et al., 2021), personal religious importance was not associated with EMI in our study. Likewise, religiosity was not linked to MFD. It could be that adding MFD made it so that religious importance did not predict group membership. Indeed, those across varying levels of personal religious importance might include those who engage in MFD, evidenced by MFD’s prevalence (i.e., 40–60% of participants in national samples report engaging in MFD; National Endowment for Financial Education, 2018; Trujillo et al., 2019). Alternatively, our measure of religiosity, a single item, could have been limited. It’s also possible that the other independent variables in the analysis were more more proximal to EMI and MFD in the SET process than religious importance.

### Limitations

In addition to the measurement limitation of moral commitment indicated above, we note several other limitations of this study. Most of our variables were single-item measures. We might have obtained more reliable measurements of the constructs if we had had multiple items measuring each.

Another limitation is that our data were cross-sectional. This is a limitation is because it means that our analyses cannot assess the direction of the associations we examined. For example, it could be that engaging the MFD led participants to have lower levels of moral commitment—not the other way around. Therefore, we cannot make any final claims about directionality with our results.

Cross-sectional data also created another limitation. The iFidelity survey did not ask participants whether their MFD or EMI behaviors had taken place in participants’ current marriage. This makes the proposition of asserting directionality even more difficult. For example, some remarried participants may have engaged in MFD and/or EMI in a previous marriage. Yet our model statistically linked their *current* marital satisfaction, moral commitment, etc. to *previous* marital betrayals. To investigate the possibility of this influencing our findings, we reran our model controlling for whether individuals were in a first marriage or in a higher order marriage. The patterns of sign and statistical significance for the associations between the main independent variables and group membership were unchanged when controlling for first versus higher-order marriage. Consequently, this temporality issue, while a problem, likely did not interfere with our findings.

Our sample was also comprised of individuals and not dyads. With dyadic data, we could have better understood if one’s own levels of our five predictors could predict a partner’s joint and separate engagement in MFD and/or EMI. Although the SET process explicitly occurs on an individual basis, understanding marital dynamics using dyadic data is generally desirable.

Finally, our sample could be tainted by selection bias. That is, those who agreed to participate in this study might have been more happy and stable couples. Additionally, the data we used was focused on marriage, but it could have been insightful to understand joint and separate occurrences of MFD and EMI by romantic relationship status (e.g., including long-term dating couples or cohabiting couples).

## Conclusion

Despite these limitations, this study adds to studies on marriage in several ways. It marks the first attempt, to our knowledge, to understand what might predict joint and separate occurrences of MFD and EMI. Both forms of marital betrayals have relationally destructive potential. Understanding their predictors and joint occurrences of MFD and EMI, then, may help professionals who work with married couples better serve their clients or even prevent some of these behaviors. Furthermore, because MFD remains understudied, discovering more information about it adds to the research that examines the money and marriage interface. Finally, in addition to generating new knowledge, our study has identified new questions regarding the phenomena of “money lies” and “extramarital ties.” The association between sex and money in adult romantic relationships remains an important topic of study.

## Data availability statement

The data analyzed in this study is subject to the following licenses/restrictions: The data are the property of the Wheatley Institute at Brigham Young University. We do not have authorization to disseminate the data. Requests to access these datasets should be directed to jason.carroll@byu.edu.

## Ethics statement

The studies involving human participants were reviewed and approved by Institutional Review Board (IRB). The patients/participants provided their written informed consent to participate in this study.

## Author contributions

JD steered the research question and data analysis and wrote the introduction, method, results, and most of the discussion sections. MS wrote much of the literature review and contributed to the discussion section. AM contributed to the literature review. All authors contributed to the article and approved the submitted version.

## Funding

JD is a Fellow of the Wheatley Institute at Brigham Young University. The Wheatley Institute funded the collection of the iFidelity data set used in this study. The work is solely the authors’. No grant numbers exist.

## Conflict of interest

The authors declare that the research was conducted in the absence of any commercial or financial relationships that could be construed as a potential conflict of interest.

## Publisher’s note

All claims expressed in this article are solely those of the authors and do not necessarily represent those of their affiliated organizations, or those of the publisher, the editors and the reviewers. Any product that may be evaluated in this article, or claim that may be made by its manufacturer, is not guaranteed or endorsed by the publisher.
